# Adult Worker Model Typologies: Examining Work–Family Policies in Fifteen European Countries

**DOI:** 10.3390/ijerph192214637

**Published:** 2022-11-08

**Authors:** Iris Po Yee Lo, Ruby Chui Man Chau, Sam Wai Kam Yu

**Affiliations:** 1 Department of Applied Social Sciences, The Hong Kong Polytechnic University, Hong Kong, China; 2 School of Sociology and Social Policy, The University of Nottingham, Nottingham NG7 2RD, UK; 3 Department of Social Work, Hong Kong Baptist University, Hong Kong, China

**Keywords:** male-breadwinner model, supported adult worker model, gender gap, labour force participation, care work, work–family policy, welfare, typology

## Abstract

This article aims to advance the discussion of government policies for improving women’s work and family life. It focuses on exploring whether it is reasonable to expect that the supported adult worker model will play an important role in guiding governments to reduce the gender employment gap and, at the same time, increase women’s resources for strengthening their control over family and work life. This model posits that governments should take a proactive approach to encouraging women to take part in formal employment, such as providing care support measures. To examine the impact of the model, this article develops an ‘input adult worker model typology’ and an ‘output adult worker model typology’ using cluster analysis of comparative data covering 15 countries. The findings show that it is important not to overestimate the impact of the supported adult worker model on reducing the gender employment gap or increasing women’s control over their lives in most of the 15 countries. The evidence generated from these typologies highlights the difficulties involved in promoting women’s welfare despite the use of the adult worker model as a substitute for the male-breadwinner model.

## 1. Introduction

Given the considerable social and economic changes witnessed during recent decades, such as a decline in fertility rates, an increase in divorce rates, and growing demand for a flexible labour force, there is a commonly held belief that the importance of the male-breadwinner model in guiding the formulation of family policy measures is in decline [1,2]. These changes have resulted in a growing volume of adult worker model studies. These studies provide an alternative perspective on how women’s family and work life should be organised. Firstly, they criticise the male-breadwinner model for depriving women of opportunities to work. They stress that all adult women and men should be given the opportunity to take part in paid employment [1,2,3,4,5,6]. Adult worker model studies are closely related to the EU’s Lisbon Strategy, agreed in 2000 and re-launched in 2005 [3]. Lisbon set the target of raising the employment rate of women to 60% by the year 2010. This increase in the female employment rate was expected to lead to a reduction in the gender employment gap. Secondly, adult worker model studies argue that the male-breadwinner model is inadequate to enable women to gain sufficient resources (such as time and financial resources) to organise their family and work life in the way they prefer [1,7]. 

This article contributes to the adult worker model literature. Its objective is to explore whether it is reasonable to expect that a widely discussed type of adult worker model, ‘the supported adult worker model’, will play an important role in guiding governments towards reducing the gender employment gap and increasing women’s access to the time and financial resources they need to strengthen their control over their family and work life. 

To meet this objective, two tasks are carried out. Firstly, we build an ‘input adult worker model typology’ and an ‘output adult model typology’ based on comparative data. Both typologies cover 15 countries (Austria, Belgium, Denmark, Finland, France, Germany, Greece, Hungary, Ireland, the Netherlands, Poland, Portugal, Spain, Sweden, and the UK) with the aim of studying diverse responses to the adult worker model and their implications for women’s family and work lives. Secondly, we use the evidence generated from the two typologies to explore three conditions that we have drawn from the adult worker model literature. We take these into consideration when developing our expectation regarding the supported adult worker model:

- Governments adopt strategies to put the adult worker model into practice.
- Governments that are committed to the extensive provision of policy measures intended to reduce the family’s costs for taking care of young children have a record of a narrow employment gap between men and women.
- Women living under the governance of governments that have a record of a narrow gender employment gap have sufficient time and financial resources to organise their own family and work lives.

As shown in the later parts of this article, the evidence provided by the input adult worker model typology informs the discussion of the first condition, and the evidence provided by the output adult worker model typology provides insights into the examination of the other two conditions. 

This article is organised into four sections. The first discusses the reasons why we should consider the three conditions when developing expectations concerning the two adult worker models. This is followed by a discussion of the methodology for developing the input adult worker model typology and the findings generated from this typology. Then we discuss the methodology for developing the output adult worker typology and the findings generated from this typology. The fourth section addresses how the findings generated from the two typologies inform the discussion of the existence of the three conditions and their implications. 

This article makes distinctive contributions to the adult worker model literature. It makes an innovative attempt to use cluster analysis to develop both the input adult worker model typology and the output adult worker model typology on the basis of comparative data. The evidence generated by these two typologies supports the analysis of the adult worker model from the input and output angles. 

### The Three Conditions Based on the Adult Worker Model Literature

A review of previous adult worker model studies provides insights into the usefulness of discussing the three conditions outlined in the introduction for the examination of the empirical significance of the supported adult worker model. Adult worker model studies show that there are two types of adult worker models—the unsupported and the supported. The unsupported adult worker model stresses that, if a government wants to encourage women to engage in the labour force, it should adopt a low intervention strategy, which is drawn from the residual welfare principle [2]. This principle stresses that the market should play a central role in the creation and allocation of wealth and that the government should only act as a secondary welfare provider. This implies that upholders of the unsupported adult worker model are expected to take only very limited actions to assist adult women to become workers [5]. 

Unlike the unsupported adult worker model, the supported adult worker model stresses that, if a government wants to encourage women to take part in formal employment, it should employ a proactive strategy, which is associated with the principle of gender equality [3]. Governments favouring this strategy are expected to actively support women to balance their labour force participation and care duties [8]. Studies show that an important way to do so is to reduce the costs of care provision borne by the family through the provision of care support measures, including subsidised childcare services, paid maternity leave benefits, and paid father-specific leave benefits [1,9,10,11,12]. If the government can provide more subsidised childcare services, the main care provider (usually a woman) will have better opportunities to reduce the time spent taking care of young children. Paid maternity leave offers women with newborn babies time resources to decide whether to spend more time taking care of their young children or to continue with their career [13]. Studies have shown that, with the support of paid maternity leave, some women choose to re-enter the job market at the end of the paid leave period, whereas others eventually give up their career [13]. Paid father-specific leave serves not only to reduce women’s responsibilities for taking care of young children but also to alleviate gender inequality in the division of care labour in the family [5,14,15]. If a government is determined to uphold the supported adult worker model, it is very likely to commit itself to the provision of these care support measures. However, if a government favours the unsupported adult worker model, its commitment to the provision of these measures should not be overestimated. 

As discussed in the introduction, previous adult worker model studies have stressed the importance of encouraging not only adult men but also adult women to take part in formal employment. In cases where a country has a record of a wide employment gap between men and women, it is reasonable to doubt its determination to put the adult worker model (of whatever type) into practice. 

The literature on the adult worker models is also concerned with whether governments’ attempts to encourage women to participate in formal employment can create favourable conditions for them to strengthen their control over their own lives [2,5]. As shown in the introduction, this concern is related to the criticisms of the male-breadwinner model. This model is based on the ideology of gender-role division between men and women, with men working full time outside the home and women responsible for domestic activities [16,17]. According to this model, a good father provides financial support for his family and may occasionally help with childcare, but the latter is not considered his major responsibility [18]. Moreover, women are expected to rely financially on their husbands’ incomes [14,19]. It is noteworthy that the male-breadwinner model can place many women in a disadvantaged position [6,20]. If women rely financially on their husbands, they are likely to have no choice but to take on most or all of the unpaid care responsibilities. Shouldering these responsibilities in turn gives women limited time to develop their own careers and consequently renders them reliant on their husbands for financial support. 

Encouraging women to take part in formal employment may enable them to tackle this Catch-22 situation. Through labour force participation, women may be able to secure sufficient financial resources to maintain a reasonable standard of living independently of their family relationships [21]. As a result, they may have more bargaining power over the allocation of unpaid work within the family, and thus gain some control over how to organise their lives. However, there is no guarantee that women can free themselves from the Catch-22 situation, even if they do participate actively in formal employment. Firstly, there has been a wide income gap between male and female workers, making it difficult for women to gain sufficient financial resources from the labour market to achieve financial autonomy within the family [6]. Secondly, it is not uncommon for female workers to still be expected to play a major role in the provision of unpaid care within the family [5,22]. For female workers, particularly those who want to develop their careers rather than taking on the major care responsibilities within the family, these traditional gendered expectations impose the burden of care work on them in addition to their paid workload. Although the possibility of some women thriving on both paid work and unpaid care work should not be ruled out, this dual burden faced by women implies that they are deprived of the necessary time to organise their lives in the ways they prefer. Thirdly, many workplaces remain male dominated in the sense that men tend to occupy top management positions and take control of decision making [20]. As a result, women may not gain sufficient power to make important decisions in the work setting. It is therefore reasonable to link studies of governments’ determination to help women challenge the Catch-22 situation with studies of governments’ determination to put the supported adult worker model into practice. 

In view of the above discussion, we should first explore whether the three conditions outlined in the introduction exist before developing any expectations about the supported adult worker model. In cases where a government does not commit itself to the provision of policy measures intended to reduce the family’s costs for taking care of young children, it is reasonable to raise doubts about its commitment to the implementation of the supported adult worker model. In cases where a government that claims to endorse the supported adult worker model has a record of a wide employment gap between men and women, it is reasonable to avoid overestimating the impact of the supported adult worker model on women’s employment. In cases where a government is successfully encouraging some women to take part in formal employment but fails to free most women from the Catch-22 situation, we should not overestimate the effectiveness of the adult worker model that is being put into practice by that government. 

## 2. Methods

### 2.1. Selection of 15 Countries 

The typologies developed in this article cover 15 countries. Comparative studies of welfare indicate that these countries do not belong to the same world of welfare capitalism because they have diverse ways of organising welfare [4,6,13,23,24]. Denmark, Finland, and Sweden are often seen as members of the social democratic group; Austria, Belgium, France, Germany, and the Netherlands are often seen as members of the conservative group; and the UK and Ireland are often seen as members of the liberal group. There is a commonly held belief that the family plays a central role in promoting people’s well-being in some Mediterranean countries, such as Greece and Spain [6,25]. Largely due to their socialist history, Hungary and Poland show different patterns of women’s participation in formal employment from those in European countries with a longer capitalist history [25]. In view of these similarities and differences, the 15 countries were chosen to be included in the development of the input and output typologies with the aim of studying diverse responses to the adult worker model and their implications for women’s family and work lives. It should be noted, however, that our selection of countries was constrained by the availability of high-quality comparable data. 

### 2.2. Selection of Variables for Developing the Adult Worker Model Typologies

Different variables were selected for developing the input and output adult worker model typologies. This sub-section describes these variables and explains the rationale for their selection. 

#### 2.2.1. Four Variables for Developing the Input Adult Worker Model Typology

The input adult worker model typology focuses on the extent of government intervention in the provision of policy measures intended to reduce the family’s costs for taking care of young children. Four variables were used to measure government intervention: the total length of paid maternity and parental leave; the length of paid father-specific leave; the out-of-pocket childcare costs for a two-earner couple family; and the out-of-pocket childcare costs for a single-parent family. 

The total length of paid maternity and parental leave refers to the total number of weeks of paid leave that can be used by the mother after the birth of a child, combining both maternity and parental leave [26]. These paid maternity and parental leave benefits give women more resources to choose whether or not to take care of their children and whether or not to maintain their career [13,27]. 

Paid father-specific leave refers to the number of paid weeks reserved for the exclusive use of fathers, including entitlements to paid paternity leave, father quotas, or periods of paid parental leave that can be used only by the father and cannot be transferred to the mother, and any weeks of paid sharable leave that must be taken by the father in order for the family to qualify for bonus weeks of parental leave [26]. The paid father-specific leave benefits may serve to reduce both the care duties borne by women and gender inequality within the family [5,27,28]. 

The out-of-pocket childcare costs for a two-earner couple family refer to the net childcare costs for a two-earner, two-child (aged 2 and 3) couple family with full-time earnings (earnings for one earner are set as equal to 100 percent of average earnings, whereas those for the other earner are set as equal to 67 percent of average earnings) [29]. Net childcare costs are calculated based on the differences between childcare fees and different kinds of childcare-related benefits, such as childcare refunds/rebates and tax reductions [29]. In other words, the net childcare costs of a family can be reduced if the government is willing to provide more childcare-related benefits for the family. 

The out-of-pocket childcare costs for a single-parent family refer to the net childcare costs for a single-parent, two-child (aged 2 and 3) family with full-time earnings (which are set as equal to 50 percent of average earnings) [29]. Similarly, these out-of-pocket childcare costs will be reduced if the government increases the level of subsidy for the family through tax reductions and/or an increase in other benefits. 

It is well documented that women’s responsibilities for taking care of young children tend to cause disruptions to their paid work [20,30]. Without the provision of policy measures that can reduce the costs of childcare borne by the family, it is reasonable to raise doubts about the commitment of a government to promoting the supported adult worker model. Although existing literature has also shown that women are likely to bear the responsibility of providing care for different family members, such as older people, adolescents, and children [31], available high-quality comparative data does not cover the provision of care for different family members in the 15 countries addressed here. Due to this limitation, the input adult worker model typology developed for this article only focuses on childcare measures. 

#### 2.2.2. Four Variables for Developing the Output Adult Worker Model Typology

The output adult worker model typology provides information about the gap between men and women in employment, the gender wage gap, the time women spend on the provision of care, and women’s opportunities to occupy decision-making positions in the work setting. 

This information sheds light on whether those governments that are keen to reduce the family’s costs for taking care of young children actually have a record of a small gender employment gap. Moreover, it provides insights into whether or not there is a positive connection between these governments’ record of a small gender employment gap and their record of providing women with better opportunities to gain sufficient financial resources to organise their lives, take on less unpaid care work within the family, and adopt a decision-making role in the workplace. 

The typology is built on four variables: the relative female labour-force participation rate, the gender wage gap, the gender gap in the time spent on cooking and/or housework, and women’s share of seats on the boards of the largest quoted companies, supervisory boards, or boards of directors. 

The relative labour force participation rate reflects the difference between the female and male labour force participation rates [28]. It is calculated as the labour force divided by the total working-age population [26]. The working-age population refers to people aged 15 to 64. 

The gender wage gap serves to show the differences in the income gained from formal employment between women and men. It is calculated as the difference between male and female median wages divided by male median wages [26]. 

The gender gap in the time spent on cooking and/or housework reflects, to a certain extent, the level of gender inequality in the domestic sphere. It also provides information about the time resources that women can mobilise and determine how to use in order to organise their lives in the ways they prefer. It is measured in terms of the percentage of men who cook and/or do housework every day and the percentage of women who cook and/or do housework every day [32]. 

The presence of women on boards provides information about the opportunities for women to make important decisions in the workplace setting. It is shown in the proportion of women on the boards of the largest quoted companies, supervisory boards, or boards of directors [32]. 

### 2.3. Statistical Analysis

Cluster analysis was used to compare and classify the countries based on the variables described above in order to develop the input adult worker model typology and the output adult worker model typology. The sources of information concerning these variables are drawn from the datasets of the OECD [26,29] and the European Institute for Gender Equality [32]. These datasets provide information covering all of the 15 countries. 

Cluster analysis is a heuristic technique designed to identify patterns of similarity and dissimilarity within a dataset [33]. It serves to measure the distance between cases along a combination of dimensions and uses this to identify groups of cases within which there is significant homogeneity and between which there is a clear boundary [34]. Hierarchical analysis is a commonly used form of cluster analysis for classifying countries, and it has been used in other research [13]. Starting with each country forming a cluster of its own, hierarchical analysis forms clusters of similar countries until all cases come together within one group [28]. This article used squared Euclidean distance and scale-standardised versions of the input and output adult worker model variables. The analysis was carried out using IBM SPSS Statistics 20, a statistical software program. 

## 3. Results

**Figure 1:**
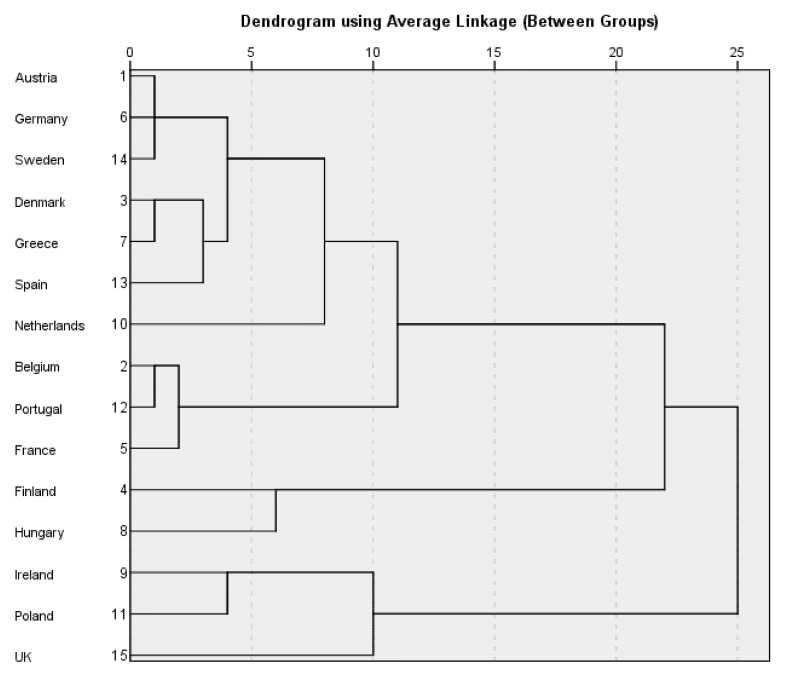
Dendrogram—Input Adult Worker Model Variables.

Table 1 outlines the unstandardised data for each of the input adult worker model variables. The results of the cluster analysis are shown in Table 2. The proximity matrix is shown in Table 3. This matrix reveals the distances between the countries when they are clustered using the four input variables. The dendrogram is shown in Figure 1. Cluster one is composed of Ireland, Poland, and the UK. These three countries have a low commitment to the provision of care support. The total length of their maternity and parental leave and the length of their father-specific leave is shorter than the average for the 15 countries. The out-of-pocket childcare costs for a single-parent family and those for a two-earner couple family are both greater than the average for the 15 countries. The members of the second cluster are Finland and Hungary. These two countries provide the longest paid maternity and parental leave (Finland, 161 weeks; Hungary, 160 weeks) among the 15 countries. The out-of-pocket childcare costs for a single-parent family are higher than those in the majority of the countries in the study. Cluster three is comprised of Belgium, Portugal, and France. The total length of their paid maternity and parental leave is shorter than that in half of the countries in the study. However, these three countries make a generous provision of paid father-specific leave. The out-of-pocket childcare costs for families are lower than the average for the 15 countries. The Netherlands is the only country in cluster four. It has a limited commitment to the provision of leave benefits. Its out-of-pocket childcare costs for a two earner-couple family are the third highest in the study. Its out-of-pocket childcare costs for a single-parent family are higher than those in the majority of countries in the study. Cluster five is the largest cluster, composed of Austria, Germany, Sweden, Denmark, Greece, and Spain. These six countries have lower out-of-pocket childcare costs for a two-earner couple family than the average for the 15 countries. All of them, except Spain, have lower out-of-pocket childcare costs for a single-parent family than the average for the 15 countries. It is important to be aware of the heterogeneity between the members of this cluster, especially in the provision of leave benefits. For example, the total length of paid maternity and parental leave in Austria is three times longer than that in Spain. As shown in the proximity matrix (in Table 3), some countries, such as Austria, Germany, and Sweden, are very similar in terms of the input variables. Austria and Germany are 0.2 in distance from each other; the distance between Germany and Sweden is 0.4, and Austria and Sweden are 0.5 apart. As shown in Table 1, these three countries have a more generous provision of leave benefits than the majority of the 15 countries. Moreover, the out-of-pocket childcare costs for the two types of family are lower than the average for the 15 countries. The proximity matrix also shows that Denmark is closer to Greece than to Spain. Denmark and Greece are 0.5 apart, but the distance between Denmark and Spain is 1.9 and the distance between Greece and Spain is 0.9. As shown in Table 1, the total length of paid maternity and parental leave in Denmark and Greece is longer than the average for the 15 countries. Both Denmark and Greece have less commitment to the provision of father-specific leave than the majority of the 15 countries. Both countries have lower out-of-pocket childcare costs for the two types of family than the average for the 15 countries. 

**Figure 2:**
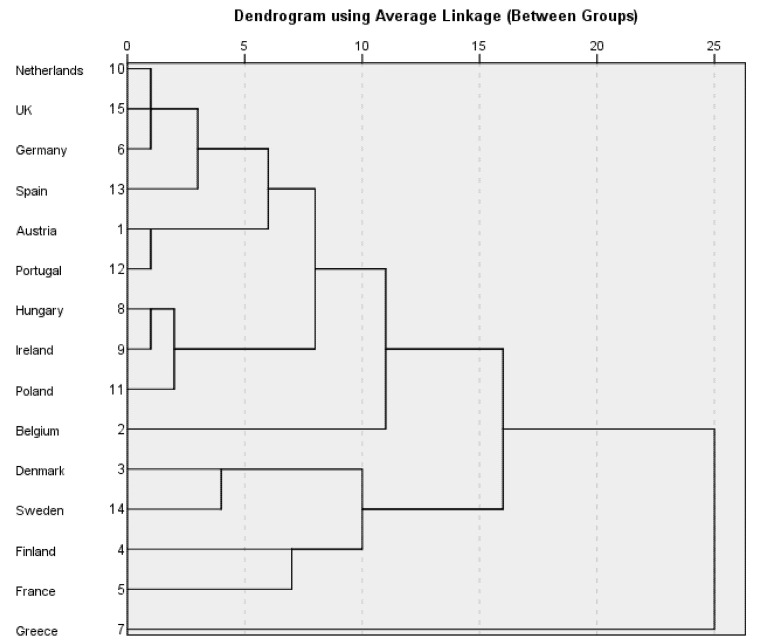
Dendrogram: Output Adult Worker Model Variables.

Table 4 outlines the unstandardised data for each of the output adult worker model variables. The results of the cluster analysis are shown in Table 5. The proximity matrix is shown in Table 6. The dendrogram is shown in Figure 2. Greece is the only country in cluster one. It has the widest gender gap in the time spent on cooking and/or housework and the lowest proportion of women on boards among the 15 countries. Cluster two is composed of Denmark, Sweden, Finland, and France. The relative labour force participation rates for these countries are lower than the average for the 15 countries. Belgium is the only country in cluster three. It has the smallest gender wage gap. Cluster four is composed of Hungary, Ireland, and Poland. The relative labour force participation rates in these three countries are higher than those in the majority of the 15 countries. Cluster five is the largest cluster, composed of the Netherlands, the UK, Germany, Spain, Austria, and Portugal. The relative labour force participation rates in these six countries are either close to or higher than the average for the 15 countries. The relative labour force participation rates in Austria and the Netherlands are the same as the average for the 15 countries. The relative labour force participation rates in Germany and the UK are higher than those in the majority of the 15 countries. The gender wage gaps in this cluster are wider than the average for the 15 countries. 

## 4. Discussion

This section discusses the key findings generated from the input adult worker model typology and the output adult worker model typology. It highlights the three conditions that we should consider when developing our expectations for the supported adult worker model and the unsupported adult worker model. This article contributes to the existing literature on the adult worker model by developing new typologies based on comparative data and advancing the analysis of the adult worker model from both the input and output angles. 

### 4.1. Condition One: Governments Adopt Strategies to Put the Adult Worker Model into Practice

The evidence generated from the input adult worker model typology shows the existence of this condition in some of the 15 countries. As shown in Table 2, there are differences between countries in their approaches to strengthening the adult worker model. 

Austria, Germany, and Sweden are more committed to the supported adult worker model, as evidenced by their relatively more generous provision of leave benefits and relatively lower levels of out-of-pocket childcare costs for families. To a certain extent, Belgium, Denmark, France, and Portugal can also be seen as upholders of the supported adult worker model. The total length of paid leave provided in Belgium and France is longer than the average for the 15 countries. Moreover, they keep the out-of-pocket childcare costs for the two types of family lower than the average for the 15 countries. In Portugal, the total length of paid leave is shorter than the average for the 15 countries. However, Portugal ranks second in providing the longest paid father-specific leave. It also keeps the out-of-pocket childcare costs for the two types of family lower than in the majority of the 15 countries. In light of their limited commitment to providing leave benefits and reducing the childcare costs for families [26,29], there is evidence to suggest that Ireland, the Netherlands, and the UK attach much more importance to the unsupported adult worker model than to the supported adult worker model. 

### 4.2. Condition Two: Governments That Are Committed to the Extensive Provision of Policy Measures Intended to Reduce the Family’s Costs for Taking Care of Young Children Have a Record of a Narrow Employment Gap between Men and Women

The evidence generated from the output adult worker model shows that this condition exists in some countries but not others. Countries such as Sweden and France, which can be considered upholders of the supported adult worker model, are marked by low relative labour force participation rates (relatively small gender differences in labour force participation) [26]. Germany provides an interesting case for the analysis of the proactive approach to the adult worker model. Similarly to Sweden and France, it can be seen as an upholder of the supported adult worker model. However, its relative labour force participation rate is higher than the average for the 15 countries. The example of Germany shows that the effectiveness of the proactive approach to strengthening women’s participation in formal employment varies between countries. It is thus important to avoid assuming that those governments that are keen to reduce the family’s costs for taking care of young children can guarantee a small employment gap between men and women. 

### 4.3. Condition Three: Women under the Governance of Governments That Have a Record of a Narrow Gender Employment Gap Have Sufficient Time and Financial Resources to Organise Their Family and Work Life

Sweden is the only country that provides a strong case for the existence of this condition. Its gender wage gap is smaller than the average for the 15 countries. It also has the smallest gender gap in the time spent on cooking and/or housework. Women’s share of seats on the boards of the largest quoted companies, supervisory boards, or boards of directors is higher than that of their counterparts in most of the 15 countries. 

However, the condition witnessed in Sweden is not supported by evidence found in other countries that are committed to upholding the adult worker model and have a smaller employment gap between men and women than the average for the 15 countries. Judging from the relative labour force participation rate [26], women in France enjoy reasonably good opportunities to take part in formal employment. However, the gender gap in the time spent on cooking and/or housework in France is wider than the average for the 15 countries. This suggests that women in France are more likely to face the dual burden of paid and unpaid work than their counterparts in most of the 15 countries. This also suggests that, even though many women in France can take an active part in formal employment, there is no guarantee that they will have sufficient bargaining power to negotiate for a reduction in their unpaid care duties within the family. In particular, for women who want to concentrate on developing their career, the wide gender gap in the time spent on cooking and/or housework implies that they are likely to be put in a difficult position by this dual burden, which hampers their chances of organising their own time in the ways they prefer [6]. The relative labour force participation rate in Denmark is lower than that in Finland and Sweden only [26]. However, it ranks only seventh among the 15 countries in terms of the proportion of women on boards. The example of Denmark indicates that increasing women’s participation in the labour market does not necessarily guarantee them opportunities to have a greater say in the workplace [32]. Portugal ranks fifth among the 15 countries for its efforts to keep the relative labour force participation rate low. However, its gender wage gap and gender gap in the time spent on cooking and/or housework are wider than those in most of the 15 countries. Moreover, women in Portugal have a much smaller share of seats on the boards of the largest quoted companies, supervisory boards, or boards of directors than their counterparts in most of the other countries. 

### 4.4. Implications for Government Approaches to Improving Women’s Work and Family Lives 

The discussion about the existence of the three conditions serves to address the central concern of this article. This concern is whether it is reasonable to expect that the supported adult worker model will play an important role in guiding the 15 countries towards reducing the gender employment gap and, at the same time, increasing women’s resources for strengthening their control over their own lives. 

It is evident from the typologies that Condition One clearly exists in a number of countries. Based on the cases of Austria, Germany, and Sweden, we should not overlook the importance of the supported adult worker model in guiding a government in formulating policies. However, the influence of this model across the 15 countries should not be overestimated, taking into account the existence (or non-existence) of the other two conditions. As shown above, those countries that provide measures to reduce the family’s costs for taking care of children do not necessarily secure a low relative labour force participation rate. Most of the countries that have a low relative labour force participation rate cannot guarantee female workers sufficient time or financial resources to strengthen their control over their own family and work lives. In view of this evidence, it is reasonable to avoid overestimating the positive impact of the supported adult worker model on government policies across the 15 countries. In line with the existing literature, our results suggest that women’s engagement in the labour market continues to be hindered by various socio-political factors, such as traditional gender norms regarding the division of domestic labour in the family and rigid gender stereotypes that undermine women’s chances of advancement into positions of power [7,35,36]. 

Further research is needed to continue to explore ways of increasing women’s opportunities to take part in formal employment and to improve their lives. As discussed earlier, the male-breadwinner model has various negative effects on women—such as depriving them of sufficient opportunities to take part in formal employment, making them financially subordinate to men, and giving them little choice but to take on unpaid care responsibilities regardless of their preferences about how to organise their family and work lives [6,20]. In order to tackle these negative effects, it is important to search for effective ways to assist women in choosing whether or not to take part in formal employment and to provide them with sufficient resources and opportunities to strengthen their control over their own lives. Therefore, more research on the supported adult worker model is needed. Alongside policies aimed at encouraging formal employment, more public education is needed to promote gender equality within and beyond the family and to eliminate negative gender stereotypes that hold women back from leadership positions [36].

## 5. Conclusions

This article has explored whether it is reasonable to expect that the supported adult worker model will play an important role in guiding governments towards reducing the gender employment gap and, at the same time, increasing women’s control over their work and family lives. It focuses on discussing the existence of the conditions that we should consider when developing this expectation. Based on the evidence generated by the two typologies developed for this article, it was found that we should avoid assuming that these conditions always exist across the 15 countries. 

The findings suggest that it is not an easy task to promote women’s well-being by replacing the male-breadwinner model with the supported adult worker model. In theory, we can reduce the employment gap between men and women by putting the supported adult worker model into practice. However, the evidence from the two typologies shows that, even though some countries attempt to support this model, there is no guarantee that they will be able to significantly narrow the gender gap in labour force participation. More importantly, even though some countries are able to increase women’s participation in formal employment, there is no guarantee that women will thus have sufficient resources to take control over their own work and family lives. 

Further efforts should be made to explore different possible types of the adult worker model as effective alternatives to the male-breadwinner model. In this study, the selection of countries and measures for the development of the typologies was constrained by the availability of high-quality comparable data. More comparable datasets of high quality are needed to facilitate further examination of how government measures can reduce the duties of the family, particularly those of women, to provide care for different family members, and how government measures can be developed to enable women to capitalise on their participation in formal employment to improve their work and family lives. 

## Figures and Tables

**Table 1:** Unstandardised data for Input Adult Worker Model Variables.

Country	Total Duration of Paid Maternity and Parental Leave (No. of Weeks) 2016	Rank	Length of Paid Father-Specific Leave (No. of Weeks) 2016	Rank	Out-of-Pocket Childcare Costs for a Two-Earner Couple Family (as % of Family Net Income) 2015	Rank	Out-of-Pocket Childcare Costs for a Single-Parent Family (as % of Family Net Income) 2015	Rank
Austria	60.0	3	8.7	6	2.6	1	6.3	8
Belgium	32.3	11	19.3	3	11.4	10	5.3	7
Denmark	50.0	7	2.0	9	9.1	8	0.0	1
Finland	161.0	1	9.0	5	17.9	12	21.5	12
France	42.0	9	28.0	1	9.8	9	3.2	4
Germany	58.0	4	8.7	6	4.7	5	1.2	2
Greece	43.0	8	0.4	13	4.1	3	5.1	6
Hungary	160.0	2	1.0	12	5.0	6	11.1	9
Ireland	26.0	13	0.0	15	26.1	14	42.1	15
Netherlands	16.0	14	0.4	13	21.3	13	11.1	10
Poland	52.0	6	2.0	9	16.3	11	32.8	14
Portugal	30.1	12	22.3	2	4.3	4	1.9	3
Spain	16.0	14	2.1	8	5.5	7	13.7	11
Sweden	55.7	5	14.3	4	3.9	2	3.4	5
UK	39.0	10	2.0	9	40.8	15	23.3	13
Mean	56.1		8.0		12.2		12.1	

Sources: OECD, 2020a [26], 2020b [29].

**Table 2:** Five Clusters: Input Adult Worker Model Variables.

Cluster 1	Cluster 2	Cluster 3	Cluster 4	Cluster 5
Ireland	Finland	Belgium	Netherlands	Austria
Poland	Hungary	Portugal		Germany
UK		France		Sweden
				Denmark
				Greece
				Spain

**Table 3:** Hierarchical cluster analysis proximity matrix (squared Euclidian distance): Input Adult Worker Model Variables.

	Austria	Belgium	Denmark	Finland	France	Germany	Greece	Hungary	Ireland	Netherlands	Poland	Portugal	Spain	Sweden	UK
Austria		2.4	1.2	8.6	5.2	0.2	1.0	5.9	14.5	5.0	6.7	2.9	1.9	0.5	15.4
Belgium	2.4		4.0	11.6	1.0	2.2	4.9	12.8	15.1	5.6	8.9	0.6	4.5	1.1	13.3
Denmark	1.2	4.0		10.4	8.4	0.8	0.4	7.0	14.1	2.7	7.3	5.5	1.9	2.2	12.3
Finland	8.6	11.6	10.4		14.2	9.5	11.3	2.9	13.4	12.2	7.4	14.8	12.9	9.7	12.7
France	5.2	1.0	8.4	14.2		4.9	9.6	16.5	21.7	11.2	14.3	0.7	9.4	2.7	19.2
Germany	0.2	2.2	0.8	9.5	4.9		1.1	6.6	16.1	4.8	8.1	2.7	2.4	0.4	15.2
Greece	1.0	4.9	0.4	11.3	9.6	1.1		7.1	13.1	3.2	6.3	6.0	0.9	2.5	13.9
Hungary	5.9	12.8	7.0	2.9	16.5	6.6	7.1		19.0	12.7	10.0	14.6	10.5	8.0	19.5
Ireland	14.5	15.1	14.1	13.4	21.7	16.1	13.1	19.0		6.4	1.8	20.5	8.9	16.8	4.3
Netherlands	5.0	5.6	2.7	12.2	11.2	4.8	3.2	12.7	6.4		3.9	9.0	2.3	6.2	4.6
Poland	6.7	8.9	7.3	7.4	14.3	8.1	6.3	10.0	1.8	3.9		12.6	4.0	8.7	5.9
Portugal	2.9	0.6	5.5	14.8	0.7	2.7	6.0	14.6	20.5	9.0	12.6		6.0	1.1	19.6
Spain	1.9	4.5	1.9	12.9	9.4	2.4	0.9	10.5	8.9	2.3	4.0	6.0		3.3	11.7
Sweden	0.5	1.1	2.2	9.7	2.7	0.4	2.5	8.0	16.8	6.2	8.7	1.1	3.3		16.4
UK	15.4	13.3	12.3	12.7	19.2	15.2	13.9	19.5	4.3	4.6	5.9	19.6	11.7	16.4	

Note: Rounded to 1 decimal place.

**Table 4:** Unstandardised data for Output Adult Worker Model Variables.

Country	Relative Labour Force Participation Rate (%) 2016	Rank	Gender Wage Gap (%) 2016	Rank	Gender Gap in the Time Spent on Cooking and/or Housework (%) 2016	Rank	Women’s Share of Members of Boards in Largest Quoted Companies, Supervisory Board, or Board of Directors (%) 2017	Rank
Austria	10.9	6	15.7	13	54.9	13	21.1	10
Belgium	10.9	6	3.7	1	48.7	12	29.6	5
Denmark	8.3	3	5.7	3	27.3	2	28.5	7
Finland	5.3	2	16.5	14	28.5	3	32.2	3
France	9.0	4	13 ^a^	9	44.0	10	42.0	1
Germany	11.0	9	15.5	12	43.2	9	30.9	4
Greece	14.8	14	4.5	2	69.3	15	9.7	15
Hungary	14.5	13	9.4	6	42.0	7	13.7	14
Ireland	13.5	12	7.4	4	40.7	6	17.3	12
Netherlands	10.9	6	14.1 ^b^	10	34.0	4	29.3	6
Poland	16.5	15	9.4	6	48.2	11	19.8	11
Portugal	10.6	5	14.3	11	59.3	14	16.3	13
Spain	11.5	11	11.5 ^b^	8	42.6	8	21.8	9
Sweden	4.8	1	8.2	5	17.5	1	36.1	2
UK	11.4	10	16.8	15	35.6	5	27.9	8
Mean	10.9		10.6		42.4		25.1	

Notes: ^a^ Data from 2015 ^b^ Data from 2014. Sources: European Institute for Gender Equality, 2019 [32]; OECD, 2020b [29].

**Table 5:** Five Clusters: Output Adult Worker Model Variables.

Cluster 1	Cluster 2	Cluster 3	Cluster 4	Cluster 5
Greece	Denmark	Belgium	Hungary	Austria
	Finland		Ireland	Germany
	France		Poland	Netherlands
	Sweden			Portugal
				Spain
				UK

**Table 6:** Hierarchical cluster analysis proximity matrix (squared Euclidian distance): Output Adult Worker Model Variables.

	Austria	Belgium	Denmark	Finland	France	Germany	Greece	Hungary	Ireland	Netherlands	Poland	Portugal	Spain	Sweden	UK
Austria		8.3	10.8	8.7	6.9	2.0	10.6	4.9	5.4	3.5	5.3	0.5	1.8	17.4	2.8
Belgium	8.3		3.5	13.6	6.7	7.1	9.0	6.3	3.6	6.7	5.8	8.5	4.1	10.8	9.6
Denmark	10.8	3.5		6.9	6.6	7.0	18.9	8.4	5.4	4.4	10.6	12.0	4.6	2.8	7.5
Finland	8.7	13.6	6.9		4.5	4.4	31.9	16.0	14.3	3.6	18.8	11.7	7.5	4.4	4.1
France	6.9	6.7	6.6	4.5		2.3	23.7	13.7	11.3	3.0	12.4	10.0	5.9	7.4	4.2
Germany	2.0	7.1	7.0	4.4	2.3		17.1	6.8	6.2	0.6	6.5	4.3	1.9	10.5	0.5
Greece	10.6	9.0	18.9	31.9	23.7	17.1		5.8	6.1	18.2	5.4	7.6	9.5	34.7	19.5
Hungary	4.9	6.3	8.4	16.0	13.7	6.8	5.8		0.5	5.8	1.1	4.5	1.9	18.9	6.4
Ireland	5.4	3.6	5.4	14.3	11.3	6.2	6.1	0.5		5.0	1.5	5.2	1.5	14.9	6.4
Netherlands	3.5	6.7	4.4	3.6	3.0	0.6	18.2	5.8	5.0		6.4	5.9	1.5	7.5	0.4
Poland	5.3	5.8	10.6	18.8	12.4	6.5	5.4	1.1	1.5	6.4		5.4	2.9	22.1	7.0
Portugal	0.5	8.5	12.0	11.7	10.0	4.3	7.6	4.5	5.2	5.9	5.4		2.5	20.2	5.3
Spain	1.8	4.1	4.6	7.5	5.9	1.9	9.5	1.9	1.5	1.5	2.9	2.5		11.1	2.2
Sweden	17.4	10.8	2.8	4.4	7.4	10.5	34.7	18.9	14.9	7.5	22.1	20.2	11.1		10.6
UK	2.8	9.6	7.5	4.1	4.2	0.5	19.5	6.4	6.4	0.4	7.0	5.3	2.2	10.6	

Note: Rounded to 1 decimal place.

## Data Availability

Publicly available datasets [26,29,32] were analysed in this study.
